# The role of lactylation in age-related diseases: a molecular perspective

**DOI:** 10.7717/peerj.20957

**Published:** 2026-05-25

**Authors:** Pengfei Li, Miaomiao Wu, Linhui Xu, Min Ji, Huaijin Guan

**Affiliations:** 1 Nantong Medical College, Nantong, China; 2 Affiliated Hospital of Nantong University, Nantong, China

**Keywords:** Lactylation, Lactate, Age-related diseases

## Abstract

Lactylation (Kla) is a novel post-translation protein modification regulation mechanism realized by the covalent coupling of lactate generated by glycolysis and lysine residues. Lactylation primarily includes histone Kla and non-histone Kla. Kla participates in biological processes in cells by regulating gene transcription and protein expression, as well as subcellular localization. Multiple organs are exposed to lactate metabolism disorder due to aging. Recent studies have demonstrated that Kla mediated by lactate metabolism disorder is involved in the occurrence and development of various age-related diseases. This article summarizes the functions of Kla in age-related diseases and the related mechanisms.

## Introduction

Aging is a complex biological process associated with the gradual decline of physiological functions and an increased susceptibility to various age-related diseases, such as neurodegenerative diseases, cardiovascular diseases, and cancers (Andersen et al., 2025; Global Nutrition Target Collaborators, 2025; Wu & Chen, 2026). With the global population aging at an unprecedented rate, age-related diseases have become a significant burden on healthcare systems and societies worldwide (Global Nutrition Target Collaborators, 2025). Therefore, understanding the molecular mechanisms underlying aging and developing effective interventions to promote healthy aging and prevent age-related diseases has become an urgent priority in biomedical research.

Lactate, as metabolite and energy source, affects the acid–base balance and metabolic pathways of the intracellular environment. Notably, senescent cells undergo metabolic reprogramming, resulting in a rapid increase in intracellular lactate content (Kawakami, Johmura & Nakanishi, 2024). Recent studies have demonstrated that lactate can serve as a donor of lactylation (Kla) of lysine residues (Fan et al., 2023a). Previous studies showed that Kla is involved in the occurrence of various diseases, including age-related diseases and tumors (Li et al., 2022b). Also, increased lactate levels further promote the formation of intracellular protein Kla, which is involved in the occurrence and development of age-related diseases by influencing protein expression and function (Du et al., 2023; Fan et al., 2023b; Pan et al., 2022b; Rho et al., 2023; Wu et al., 2023; Zhang et al., 2023b).

Given the rapid aging of the global population and the increasing prevalence of age-related diseases, a deeper understanding of lactylation’s role in aging is not only scientifically compelling but also clinically urgent. In this review, we aim to summarize the current knowledge of lactylation in age-related diseases, highlighting its functions and underlying mechanisms. It is our hope that this review will stimulate further research into this important area and contribute to the development of novel therapeutic strategies for promoting healthy aging and treating age-related diseases.

## Survey Methodology

Data were searched from the PubMed (https://pubmed.ncbi.nlm.nih.gov/), CNKI (https://www.cnki.net/) databases. The keywords used were as follows: “aging”, “age-related diseases”, “neurodegenerative diseases”, “cardiovascular diseases”, “cerebrovascular diseases”, “osteoporosis”, “age-related lung diseases”, “age-related hepatic diseases”, “lactylation”, “lactate”, “Histone lactylation”, “Non-histone lactylation”. Inclusion criteria: The content of the literature involved the metabolism of lactate, the classification and influencing factors of lactylation, and the effect of lactylation modification on aging and related diseases, the mechanisms underlying aging and related diseases, and the specific mechanisms through which lactylation is involved. Exclusion criteria: low quality literature not related to the topic of this paper. A total of 116 articles were included. We carefully analyzed and summarized the extracted key information, followed by a systematic introduction and in-depth discussion.

### Lactic acid metabolism

Lactate exists in two isomeric forms: L-lactate and D-lactate. Nevertheless, L-Lactate predominantly occurs in the human body, where it is primarily generated through the action of lactate dehydrogenase A (LDHA), which catalyzes the reduction of pyruvate into lactate (Li et al., 2022b). Lactate is a classical by-product of glucose metabolism, and its production pathway largely depends on glycolysis (Li et al., 2022b; Zhang et al., 2019). Glucose is transferred from the extracellular space to the cytoplasm under physiological conditions, then converted into pyruvate and two adenosine triphosphates (ATPs) through various glycolytic enzymes. The pyruvate is transported to the mitochondrion under aerobic conditions for the tricarboxylic acid cycle and oxidative phosphorylation (OXPHOS), generating about 32 ATPs (Chen et al., 2021; Yang et al., 2024). Under hypoxic conditions, OXPHOS is blocked, and pyruvate is converted to lactate by LDHA, with the early steps of glycolysis producing 2 ATP molecules when glucose is converted to pyruvate, while the conversion of pyruvate to lactate serves only to regenerate NAD+ to sustain glycolysis. Nevertheless, large amounts of lactate are generated in tumor cells and some other proliferating cells, and even in oxygen-enriched environments *via* the glycolysis pathway to obtain energy, a phenomenon known as the “Warburg effect” (Brown & Ganapathy, 2020). Under hypoxic conditions, the oxidation of NADH to NAD^+^ by LDHA is a prerequisite for maintaining glucose oxidation. Since NAD^+^ is an essential cofactor for the glyceraldehyde - 3 - phosphate dehydrogenase (GAPDH) reaction, glycolytic flux will be interrupted if there is no continuous regeneration of NAD^+^ (Mathew et al., 2024). Overall, intracellular oxygen content, mitochondrion OXPHOS, and the glycolysis pathway influence lactate production.

The regulation of lactate homeostasis in cells also depends on the solute carrier family composed of monocarboxylate transporters (MCTs) 16. To date, 14 MCTs family proteins have been identified in various tissues. MCTs play important roles in cellular nutrient transport, cellular metabolism, and pH regulation. Among them, MCT1-4 are major lactate transporter proteins. For example, MCT1 is highly expressed in red blood cells and endothelial cells, exhibits high affinity for lactate and pyruvate, and requires the auxiliary protein CD147 for proper function (Li, Nguyen & Bonanno, 2014; Luz et al., 2017). MCT2 has a high affinity for lactate, and its function is dependent on the chaperone protein Embigin (GP70) (Kirat & Kato, 2015; Sivaprakasam et al., 2017). MCT3, primarily located in the retinal pigment epithelium (RPE), has the highest affinity for pyruvate and functions without the need for auxiliary proteins (Deora et al., 2005; Kirat et al., 2009; Sivaprakasam et al., 2017). MCT4, mainly expressed in tissues with high glycolytic activity such as skeletal muscle and white blood cells, has a higher affinity for lactate than MCT1 and, similar to MCT1 and MCT2, also interacts with CD147 (Iizuka, Machida & Hirafuji, 2014). These distinct characteristics enable MCT1-4 to synergistically facilitate lactate shuttling between glycolysis cells and oxidized cells, thereby maintaining intracellular lactate equilibrium (Bonen, Heynen & Hatta, 2006; Felmlee et al., 2020; Halestrap, 2013). Nevertheless, the roles of other MCTs in lactate is unclear.

### Types of protein Kla

The nucleosome is the basic structural unit of chromatin, consisting of a segment of DNA wrapped around a histone octamer (comprising two copies each of H2A, H2B, H3, and H4). Histone is a key component of chromatin, playing an important role in the regulation of gene expression (Zhang et al., 2023d). Post-translational modification of histone is essential for the regulation of gene expression and chromatin structure (Lu et al., 2021; Sadakierska-Chudy & Filip, 2015). For instance, acetylation and methylation of histone are often associated with gene activation or silencing (Gong & Miller, 2019; Grunstein, 1997; Koprinarova, Schnekenburger & Diederich, 2016; Schaft et al., 2003). Zhang et al. (2019) first proposed Kla, a novel post-translational modification of histone, in 2019 and found that it can promote gene transcription. Also, 28 core histone Kla sites, including H2BK5, H3K18, H3K23, H4K5, H4K8, and H4K12, have been identified in human and mouse macrophages *via* protein mass spectrometry. Besides, several novel histone modification sites have been identified. (H3K9, H3K56, H3K14, H2BK6, H4K80 and H2AK4) (Dai et al., 2023; Pan et al., 2022a; Sun, Chen & Peng, 2022; Xu et al., 2023; Yin et al., 2023). Additionally, the study of upstream regulatory mechanism of histone Kla has revealed that: (1) Inflammation, hypoxia, fine particulate matter in the air (PM2.5), and other factors can stimulate glycolysis pathway activation, promote lactate generation, and lead to histone Kla in gene promoter zone (Li et al., 2024a; Li et al., 2022a; Lin et al., 2024); (2) glycolysis related proteins (*e.g.*, GBM derivatives, NR4A3, HSPA12A, IGF2BP2, GLUT3, STAT5) can increase glycolysis rate, accelerate lactate generation, thus increasing histone Kla (De Leo, Ugolini & Veglia, 2024; Huang et al., 2023; Ma et al., 2024; Yang et al., 2023; Yu et al., 2024; Zhou et al., 2024); (3) Dux proteins affect histone Kla by interacting with Kla enzymes (Hu et al., 2024). Overall, histone Kla contains site modifications and can affect the transcriptional activity of the target gene, thus participating in cellular life activities.

The first study involving Kla omics analysis of fungal Botrytis cinerea proteins identified 273 Kla sites in 166 Kla proteins, of which 62% of the proteins contained only one Kla site, while 38% had multiple Kla sites. Further subcellular localization analysis found that most Kla proteins are distributed in the nucleus (36%), with a similar proportion in the mitochondrion (27%) and cytoplasm (25%) (Gao, Zhang & Liang, 2020). Additionally, 387 Kla sites have been identified in 257 Kla proteins in African Trypanosoma brucei, of which 76% of the proteins had one Kla site, 14% had two Kla sites, and 10% had three or more Kla sites. Subcellular localization identified 38% of Kla proteins in the nucleus, 35%in cytoplasm (35%), and 11% in the mitochondrion (11%) (Zhang et al., 2021a). Similar results were found in Toxoplasma gondii (Zhao et al., 2022). Besides non-histone Kla in pathogenic microbe, 114 Kla sites have been found in 62 Kla proteins of renal epithelial cells 293T, of which 63% contained single Kla sites and 37% had multiple Kla sites. Subcellular localization showed that the proteins were mainly distributed in the cytoplasmic components, of which most had dual subcellular localization (Sun, Chen & Peng, 2022). Overall, although most Kla proteins have one Kla site in eucaryotes and prokaryotes, the subcellular distribution of the modified proteins is diversified. Furthermore, Kla proteins in prokaryotes are mainly distributed in the nucleus, while those in eukaryotes are mainly distributed in the cytoplasm (Table 1).

**Table 1:** Lactylation of proteins from different species.

**Species**	**Kla proteins**	**Kla sites**		**Subcellular distribution**				**References**
		One	Two or more	Nucleus	Cytoplasm	Mitochondrion	Others	
Fungal pathogens	166	62%	38%	36%	25%	27%	12%	Gao, Zhang & Liang (2020)
Toxoplasma gondii	523	60%	40%	36.9%	26%	16.83%	20.27%	Zhao et al. (2022)
Mammals	62	63%	37%	11%	88%	/	/	Sun, Chen & Peng (2022)
African Trypanosoma brucei	257	76%	24%	38%	35%	11%	16%	Zhang et al. (2021a)

Kla is involved in various cellular functions, such as energy metabolism, gene regulation and protein biosynthesis, RNA splicing, nucleosome assembly, and DNA damage repair by regulating protein expression and functional activity (Chen et al., 2024; Gao, Zhang & Liang, 2020; Sun, Chen & Peng, 2022; Zhang et al., 2021a; Zhao et al., 2022). Meanwhile, Kla can change the subcellular localization of proteins, shifting them from the nucleus to the cytoplasm to play different regulatory roles. Besides, Kla has competitive crosstalk with various post-translational modifications (Li et al., 2024b; Yang et al., 2022b; Zhang et al., 2024b). Also, Kla can increase the binding on the target gene of the transcription factor, enhancing its activity in promoting self-renewal of embryonal stem cells (Dong et al., 2024). Nevertheless, the mechanism of action of Kla is unclear.

### Factors influencing Kla

The regulation of lactylation modification is influenced by a multitude of factors, including glycolysis and the interaction between acyltransferases, which lactyl-based acyltransferase, and protein post-translational modifications, ultimately impacting the extent of lactylation modification in proteins.

Lactate is a product of glycolysis. *In vitro* use of OXPHOS inhibitors rotenone and 2-deoxy-D-glucose (2-DG, a glucose analogue) activates and inhibits glycolysis, thereby regulating intracellular lactate production, thus leading to the occurrence of Kla (Fan et al., 2023b; Gu et al., 2022; Yu et al., 2021; Zhang et al., 2019). LDH is also a key oxidoreductase in the glycolysis pathway, catalyzing the reduction of pyruvate to lactate, which is mainly classified into LDHA and lactate dehydrogenase B (LDHB) (Fondy & Kaplan, 1965; Markert, Shaklee & Whitt, 1975). The inhibition of protein expression and functional activity of LDHA and LDHB significantly suppress lactate and Kla levels (Yang et al., 2022a; Zhang et al., 2019; Zhang et al., 2021a; Zhang et al., 2023b). The inhibition of LDHA and LDHB expression decreases Histone Kla levels in human renal clear cell adenocarcinoma cells and human renal carcinoma cells, especially after LDHA inhibition (Yang et al., 2022a). This indicates that the sensitivity and specificity of LDHA to intracellular Kla vary in different diseases.

**Figure 1:**
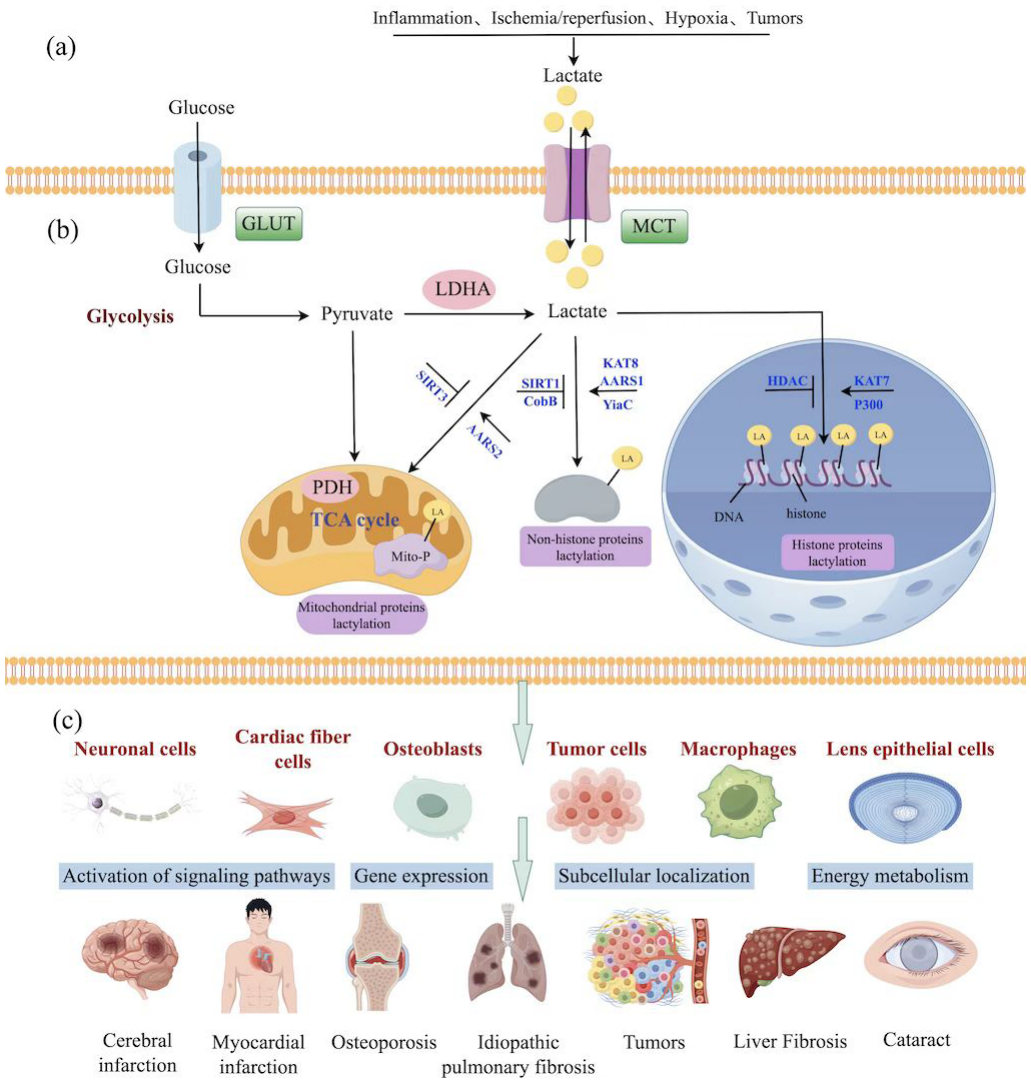
The progression of Kla in age-related diseases. (A) The influencing factors of lactate production. (B) Protein Kla and its modifying enzyme at different sites. (C) Mechanisms of action of Kla in different age-related diseases.

Lactate is a key component in the glycolysis pathway (Fig. 1A). The resulting Kla also provides negative feedback to regulate glycolysis. Pan et al. (2022b) found that activation of the glycolysis pathway can promote the production of large amounts of lactate in patients with neurodegenerative diseases (NDs) and animal models. Lactate promotes the development of Kla in histone H4, which increases gene expression of pyruvate kinase isozyme type M2 (PKM2, a key enzyme in glycolysis). PKM2 provides positive feedback to promote the activation of the glycolysis pathway. Overall, the intracellular glycolysis pathway directly affects the occurrence of lactate and Kla. The occurrence of Kla and further mechanism regulation can be controlled through targeted intervention of key enzymes in glycolysis.

Kla is a dynamically changing type of post-translation protein modification (Fig. 1B). Besides, current research mainly focused on identifying the identity and function of the writer and eraser enzymes required for Kla remains. To date, histone acetyl transferases (HATs) and histone deacetylases (HDACs) are the widely recognized modifying enzymes (Xu et al., 2021; Zhang et al., 2019). The HAT family of Kla-promoting proteins mainly includes the p300/CREB binding protein (p300/CBP) family, the GCN5-related N-acetyltransferase (GNAT) family, and the Lysine Acetyltransferase (KAT) family. The modifying effect is mainly exerted through p300/CBP. Overexpression/knockdown of p300 protein in HCT116 and HEK293T cells of patients with colon cancer can significantly change intracellular histone Kla levels (Zhang et al., 2019). However, p300 inhibitor C646 can also inhibit Kla upregulation caused by p300 overexpression (Cui et al., 2021; Yang et al., 2022b). These findings putative that p300 is a Kla writer. Meanwhile, the lactyl donor provided by p300 is about 1,000-fold lower than that of acetyl coenzyme, indicating that p300 may not be a lactyl transferase (Ju et al., 2024). A recent study involving modified omics analysis of Kla proteins in *E. coli* revealed that the YiaC protein, belonging to the GNAT family, can also induce lysine lactyl-based transferase activity (Dong et al., 2022). Additionally, the KAT family proteins, HBO1 (KAT7) and MOF (KAT8), can act as lactyl transferase to catalyze histone Kla. Furthermore, HBO1 mainly catalyzes the occurrence of Kla at the histone H3K9 site. Besides, HBO1 requires various auxiliary proteins, such as JADE1 and BRPF2, to promote histone Klamodifying enzyme activity (Niu et al., 2024). Notably, MOF enhances the translation rate of intracellular proteins by promoting the Kla of translation initiation growth factor (eEF1A2), leading to tumor development (Xie et al., 2024). Nonetheless, this is an emerging research field, and many lactyl transferases require further analysis.

Alanyl-tRNA synthetase 1 (AARS1) possesses lactyl-based transferase activity. AARS1 can directly utilize lactate and ATP to catalyze Kla modification of the critical complex YAP/TEAD in the Hippo signaling pathway. AARS1, as a lactate sensor and lactate transferase, can bind to lactate and catalyze the formation of lactate-AMP, then transfer lactate to lysine receptor residues for Kla modification. Alanyl-tRNA Synthetase 2 (AARS2) is also Kla writer in the mitochondrion. AARS2 is a protein lysine lactyl-based transferase. Hypoxia induces accumulation of AARS2, resulting in Kla of lysine sites of pyruvate dehydrogenase complex and carnitine palmitoyl transferase 2 (CPT2) (Mao et al., 2024). While AARS1 facilitates lactylation through a lactyl-AMP intermediate (Mrozikiewicz et al., 2024), another critical pathway involves the generation of L-lactyl-CoA as the direct acyl donor for enzymatic lysine lactylation. Recent biochemical evidence suggests that acyl-CoA synthetase short-chain family member 2 (ACSS2), traditionally known for its role in acetyl-CoA synthesis, plays a pivotal role in this process. ACSS2 can catalyze the ligation of L-lactate with coenzyme A (CoA) to form L-lactyl-CoA in an ATP-dependent manner (Mrozikiewicz et al., 2024; Zong et al., 2025). This high-energy thioester then serves as a substrate for histone acetyltransferases (KATs), most notably p300/CBP, which transfers the lactyl group onto the *ɛ*-amino group of lysine residues (Zong et al., 2025). However, it is noteworthy that the catalytic efficiency of p300 for lactylation is significantly lower than for acetylation, suggesting that high local concentrations of L-lactyl-CoA are required (Mrozikiewicz et al., 2024). The identification of the ACSS2-Lactyl-CoA-p300 axis provides a mechanistic explanation for how metabolic lactate fluctuations are transduced into epigenetic signals, distinguishing it from the non-canonical AARS1-mediated pathway. However, further research should assess whether AARS1 and AARS2 can induce Kla of mitochondrion protein (Ju et al., 2024; Zong et al., 2024). A study (2019) (Moreno-Yruela et al., 2022) showed that Kla erasers include histonedeacetylase 1-3 (HDAC1-3) and sirtuin 1-3 (SIRT1-3), which are currently the most potent *in vitro* eraser protein (Moreno-Yruela et al., 2022). A recent study also indicated that CobB can delactylate enzymes in *E. coli* (Dong et al., 2022). Nonetheless, future studies should assess other potential Kla enzymes.

Proteins are exposed to multiple post-translational modifications, including ubiquitination, methylation, and acetylation. These modifications affect each other, where one post-translational modification acts by intervening in the formation of another post-translational modification or in a synergistic manner. This phenomenon is known as crosstalk of post-translational modification (Hunter, 2007). Histone Kla is closely related to acetylation (Tian & Zhou, 2022; Yang et al., 2022b; Zhang et al., 2019). The acetylation level of histone decreases when histone Kla increases because of the high distributional similarity between histone Kla and histone acetylation, suggesting a competitive relationship (Zhang et al., 2019). Additionally, the two bind in a competitive manner to modify the lysine residues of histone H3, thereby regulating the expression of a specific set of genes (Hagihara et al., 2021). Besides, Kla downregulation increases the phosphorylation of proteins in this region, leading to disease development (Zhang et al., 2024b). Also, Kla binds competitively to ubiquitination. Specifically, Kla of the E3 ligase NEDD4 affects Caspase-11 ubiquitination and degradation by altering its binding affinity to the substrate protein Caspase-11 (Li et al., 2024b). Notably, a crosstalk regulatory mechanism exists between histone Kla and RNAm6A methylation in the pathogenesis of ocular melanoma (Fu et al., 2022; Yu et al., 2021). In summary, the lactate-induced modification of proteins can regulate both the expression and function of proteins through interactions with various post-translational modifications. Furthermore, gene transcription may be modulated by integrating epigenetic regulatory mechanisms, such as RNA methylation (Table 2).

**Table 2:** Types and effects of modifications of lactylation modification crosstalk.

	**Modification**	**Mechanism**	**References**
**Lactylation**	Acetylation	Lactate stimulates HMGB1 acetylation	Yang et al. (2022b)
		Histone lactylation increases histone acetylation decreases during polarization of M1 macrophages.	Zhang et al. (2019)
	Ubiquitination	NEDD4-K33 lactylation inhibited Caspase11 ubiquitination.	Li et al. (2024b)
	RNA Methylation	Lactylation on zinc-finger domain of METTL3 enhances its capture of m6A-modified RNA.	Xiong et al. (2022)
		Histone lactylation promoted YTHDF2 to recognized PER1 and the m6A site of TP53 mRNA binding for RNA degradation.	Yu et al. (2021)

### Role of Kla in neurodegenerative diseases

Neurodegenerative diseases (NDs), including Alzheimer’s disease (AD), Parkinson’s disease (PD), and Huntington’s disease (HD), are one of the most common age-related neurodegenerative brain diseases. Amyloid-β (Aβ) plaques and deposition of Tau nerve fibers are typical pathologic features of AD (Miller et al., 2022). Microglia are immune cells resident in the central nervous system (CNS) that provide immune protection under pathophysiological conditions. Microglia participate in the clearance of Aβ proteins, which usually accumulate around amyloid plaques, regulating the occurrence and development of AD. Nevertheless, the monitoring and clearance functions of microglia are gradually lost with the aging of the body and the progression of multiple NDs (Mawuenyega et al., 2010). Although the specific causes of this microglia dysfunction are unclear, the inflammatory activation process of microglia may be related to dysfunction (Fagan et al., 2021; Tejera et al., 2019).

Recent studies have demonstrated that pro-inflammatory activation of microglia is a hallmark of AD, which involves a switch from OXPHOS to glycolysis (Hu et al., 2020; McIntosh et al., 2019). Notably, glycolysis metabolite lactate levels are significantly increased in the cerebrospinal fluid and hippocampus of AD mice (Liguori et al., 2015). Histone H4K12la modification levels are significantly increased in brain tissues of AD clinical patients and mice with increased accumulation of lactate. Additionally, H4K12la can activate the transcription of the glycolysis-related gene PKM2 in microglia, forming glycolysis/H4K12la/PKM2/glycolysis circuit. This further exacerbates glucose metabolism disorders in AD patients, leading to microglia dysfunction. The inhibition of this circuit significantly reduces Aβ protein accumulation and improves spatial learning and memory ability in AD mice (Pan et al., 2022b). Therefore, H4K12laKla caused by microglia glycolysis pathway abnormality may be a key factor leading to microglia pro-inflammatory activation and dysfunction.

Although senescent microglia play an important role in AD pathogenesis, the exact mechanism is unclear. Protein modification mass spectrometry is widely used to detect an increase in the modification level of the histone H3K18la site in microglia and hippocampus tissues of naturally senescent mice and AD mice. The up-regulated H3K18la activates the NF-*κ*B signaling pathway by significantly binding to Rela (p65) and NF*κ*B1 promoter (p50), up-regulating the aging-related secretory phenotypes of IL-6 and IL-8, thereby promoting the aging and AD phenotypes (Wei et al., 2023). Overall, histone Kla influences the functional state of microglia by regulating the transcriptional activity of genes, thereby participating in the occurrence and development of AD (Fig. 1C).

### Role of Kla in cancer

Cancer is fundamentally an age-related disease, with its incidence rising sharply as a consequence of accumulated cellular damage and age-associated metabolic decline. A hallmark of both aging tissues and malignant tumors is metabolic reprogramming, characterized by a shift from oxidative phosphorylation to glycolysis—a phenomenon that leads to the massive accumulation of lactate within the microenvironment (Yue et al., 2025) Recent evidence suggests that this “lactate-rich” landscape, often exacerbated by the metabolic symbiosis between aging stromal cells and tumor cells, serves as the primary driver for Kla, a kaleidoscopic post-translational modification that fuels the metastatic cascade (Yue et al., 2025; Zhang et al., 2019).

In the context of aging, senescent cells in the tumor microenvironment (TME), such as cancer-associated fibroblasts (CAFs), contribute to an elevated glycolytic flux, raising L-lactate levels from a physiological 2–3 mM to upwards of 40 mM in tumor tissues (Yue et al., 2025). This age-dependent lactate accumulation facilitates histone Kla, primarily through the activity of “writers” like p300 and HBO1. For instance, p300-mediated H3K18la has been shown to activate the transcription of profibrotic and oncogenic genes, such as c-Myc and SRSF10, which promote proliferation and spheroid formation in breast cancer (Pandkar et al., 2023; Yue et al., 2025). Similarly, HBO1-mediated H3K9la drives pathways involved in cancer progression, suggesting that the epigenetic “memory” of a high-lactate environment can permanently alter gene expression patterns in aging tissues (Kotha et al., 2024).

Beyond histones, non-histone protein lactylation plays a critical role in bypassing age-related tumor suppression. A pivotal example is the lactylation of the “guardian of the genome”, p53. The alanyl-tRNA synthetase AARS1 acts as a lactate sensor and lactyltransferase, modifying p53 at specific lysine residues (K120 and K139). This modification prevents p53 from binding to DNA, thereby inhibiting its ability to induce cell cycle arrest or apoptosis—mechanisms that are vital for preventing cancer in aging organisms (Wu et al., 2024; Zong et al., 2025). Furthermore, in colorectal cancer, the lactylation of METTL3 by p300 enhances RNA m6A modification, leading to immunosuppression by promoting the polarization of tumor-infiltrating myeloid cells (Jeurissen et al., 2022).

Ultimately, the “lactylation wave” in cancer represents a convergence of metabolic stress and epigenetic rewiring. As tissues age, the dysregulation of lactate homeostasis creates a permissive environment for Kla-mediated activation of oncogenic pathways and the evasion of immune surveillance. Targeting the enzymes involved in this process, such as the ACSS2-KAT2A complex or the MCTs, offers a significant therapeutic opportunity to disrupt the link between aging, metabolism, and cancer.

### Role of Kla in the pathogenesis of cataracts

Recent advancements in ocular metabolomics and epigenetics have redefined the role of lactate from a metabolic byproduct to a central regulator of lens homeostasis. While the lens relies on glucose metabolism to maintain transparency, aging and metabolic disorders like diabetes mellitus significantly disrupt these pathways, leading to the accumulation of lactate and the subsequent induction of protein Kla (Li et al., 2025; Zhou et al., 2025).

A pivotal study by Li et al. (2025) utilized spatial metabolomics to demonstrate a significant disparity in lactate distribution within the lenses of patients with diabetic cortical cataracts (DCC). Lactate levels were found to be markedly higher in the opacity zones (OZ-LFCs) compared to the clear zones (CZ-LFCs) (Li et al., 2025). This localized lactate accumulation serves as a precursor for both histone and non-histone lactylation, which fundamentally alters the biological functions of human lens epithelial cells (HLECs). Specifically, transcriptomic and proteomic profiling identified 591 H3K18la-modified genes and 953 lactylated proteins in HLECs, including key metabolic and structural regulators such as Pyruvate Kinase M2 (PKM2) and Nucleophosmin 1 (NPM1) (Li et al., 2025). Mechanistically, elevated lactate levels exacerbate high-glucose-induced lens opacification by triggering HLEC apoptosis and impairing DNA damage repair mechanisms through these lactylation-mediated epigenetic shifts (Chai et al., 2025; Li et al., 2025).

The significance of the lactate-lactylation axis extends across various ocular pathologies. As highlighted by Zhou et al. (2025), the “Retinal Warburg Effect” produces substantial lactate even under normoxic conditions, and while this supports physiological functions in a healthy state, its dysregulation is a pathological trigger for several eye disorders (Zhou et al., 2025). In the context of cataracts, the interplay between metabolism and epigenetics suggests that lactylation acts as a molecular bridge, linking systemic metabolic stress to localized lens fiber degradation (Chai et al., 2025; Zhou et al., 2025). Furthermore, Chai et al. (2025) emphasize that the meticulous regulation of glycolytic enzymes, such as LDHA, is paramount in orchestrating ocular morphogenesis and maintaining homeostasis. The targeted modulation of lactate/lactylation signaling, therefore, emerges as a promising therapeutic strategy to ameliorate the progression of vision-impairing conditions like cataracts (Chai et al., 2025).

In conclusion, the discovery of the lactate-lactylation-HLEC axis provides a novel epigenetic perspective on cataract formation. By fine-tuning these pathways—specifically by inhibiting excessive glycolysis or targeting specific lactylation “writers” like p300—it may be possible to preserve lens transparency and restore visual acuity in aging populations.

### Role of Kla in cerebrovascular diseases

Cerebral infarction (CI) is a common vascular disease caused by a sudden decrease or blockage of cerebral blood flow (Sun et al., 2020). Blood vessels become progressively more fragile and vulnerable with aging, significantly increasing the risk of cerebrovascular stroke (Carrillo et al., 1991; Sacco et al., 1995; Tanaka et al., 1993). Cerebral ischemia-reperfusion (CI/R) is associated with energy metabolism disorder (Zhang et al., 2021b; Zhao et al., 2021). Physiologically, neurons have low levels of glycolysis. However, CI inhibits aerobic respiration of neurons, increases glycolysis, and exacerbates disease progression (Zhao et al., 2021). The increased lactate of glycolysis promotes the occurrence of Kla in lymphocyte cytosolic protein 1 (LCP1). LCP1 is an actin-binding protein, which is significantly up-regulated in the CI/R model (Wen et al., 2019). LCP1Kla levels are significantly increased *in vitro* and *in vivo* CI models. Also, the inhibition of glycolysis reduces LCP1Kla, leading to the degradation of LCP1 to mitigate CI progression (Zhang et al., 2023c). Additionally, the lactate-modifying enzyme LDHA regulates HMGB1 transcription and expression in middle cerebral artery occluded rats and oxygen and glucose-deprived/re-oxygenated N2a cells by increasing the enrichment of H3K18la in the promoter region of HMGB1. This induces pyroptosis, leading to CI/RNP (Yao & Li, 2023). These findings reveal that Kla may be involved in CI exacerbation.

### Role of Kla in cardiovascular diseases

Myocardial infarction (MI) is caused by persistent ischemia of the heart or coronary arteries and has a high mortality rate (Martin et al., 2024). The end-stage pathology of MI is characterized by ventricular remodeling, which is mainly associated with myocardial fibrosis. Myocardial fibrosis involves pathological ECM remodeling and fibroblast activation and is a key indicator of cardiac aging, leading to myocardial structural disorders and cardiac dysfunction. Endothelial-to-mesenchymal transition (EndoMT) is a key mechanism for the differentiation of vascular endothelial cells into fibroblasts.

EndoMT occurs in endothelial cells, where a series of cellular and molecular changes alter phenotypes of mesenchymal cells (myofibroblasts) (Kovacic et al., 2019). Cardiac hypoxia after MI promotes EndoMT, leading to myocardial fibrosis (Tombor et al., 2021). Studies have demonstrated that transforming growth factor β (TGF-β) plays a key role in EndoMT (Goumans & Ten Dijke, 2018). Activation of TGF-β signaling promotes the expression of EndoMT-related transcription factors (Kokudo et al., 2008). Snail1 is a transcriptional factor of TGF-β that plays an important role in regulating EMT. High levels of lactate are positively associated with poor prognosis and mortality rates in cardiac patients (Biegus et al., 2019; Zymlinski et al., 2018). Furthermore, lactate modifying enzyme CBP/p300 binds to Snail1 in the MI mouse model, leading to Kla of Snail1 through MCT-dependent signaling, which mediates EndoMT after MI, further exacerbating myocardial fibrosis (Fan et al., 2023b). Next, this study observed that the administration of lactate facilitated the nuclear translocation of Snail1 under hypoxic conditions. Chromatin immunoprecipitation (ChIP) assays revealed that nuclear Snail1 binds to the TGFB1 gene, thereby promoting the expression of TGF-β. Then, the inhibition of Snail1 attenuated both EndoMT and the activation of the TGF-β/Smad2 pathway in the presence of lactate following hypoxia, highlighting the role of Snail1 in lactate-induced, TGF-β/Smad2-mediated EndoMT.

Besides, post-MI triggers a complex inflammatory cascade, which is critical for acute injury and post-infarction repair (Nahrendorf & Swirski, 2016). The persistent excessive inflammatory response after MI exacerbates myocardial injury and cardiac dysfunction. Moreover, mononuclear-macrophages characterized by reparative genes can counteract the inflammatory response and promote cardiac repair (Hilgendorf et al., 2014). A study discovered that monocytes undergo metabolic changes early in MI, with altered glycolysis and MCT1-facilitated lactate transport enhancing histone H3K18 lactylation (Wang et al., 2022). They also identified IL-1β-driven GCN5 recruitment as a catalyst for histone H3K18 lactylation, acting as a regulatory element for monocyte histone lactylation and repair genes (Lrg1, Vegf-α, and IL-10) expression after MI. Ultimately, histone lactylation aids in early activation of reparative gene expression in monocytes, crucial for immune balance and cardiac repair post-MI (Wang et al., 2022).

Lactate is a key energy substrate of the heart (Karwi et al., 2019; Murashige et al., 2020). Lactate plays a protective role in cardiac hypertrophy, myocardial injury and heart failure (Bosso et al., 2021; Cluntun et al., 2021; Dai et al., 2020; Haege, Huang & Huang, 2021; Nalos et al., 2014). A recent study demonstrated that α-myosin heavy chain (α-MHC) is lactylated at lysine 1897, affecting its interaction with Titin. In α-MHC K1897R knock-in mice, loss of lactylation weakens α-MHC-Titin interaction and cardiac performance (Zhang et al., 2023b). In addition, p300 and Sirtuin 1 are identified as the acyltransferase and delactylase for α-MHC. Lowering lactate levels decreases α-MHC lactylation and worsens heart failure, while increasing lactate enhances lactylation and improves heart function. Therefore, α-MHC lactylation is crucial for cardiac health, with reduced lactate levels during myocardial injury leading to decreased lactylation (Zhang et al., 2023b). Overall, Kla regulates cardiac function by regulating EndoMT, anti-inflammatory and pro-angiogenic mechanisms.

### Role of Kla in osteoporosis

Osteoporosis is caused by an imbalance between osteoblast bone formation and osteoclast bone resorption (Sanghani-Kerai et al., 2018). The differentiation ability of bone marrow mesenchymal stem cells (BMSCs) to osteoblasts gradually decreases with aging, leading to the occurrence of osteoporosis (Morrison & Scadden, 2014). The transformation between osteoblasts relies on the oxygen and nutrient supply of endothelial cells (ECs) (Eelen et al., 2018). Compared with other cells, ECs produce large amounts of ATP through glycolysis. Angiogenesis stimulation further up-regulates EC glycolysis to maintain angiogenesis (Potente & Carmeliet, 2017). In one study, 86 serum samples were collected from osteoporosis and non-osteoporosis patients, and metabolomics results confirmed that lactate level in serum decreases in osteoporosis patients (Wu et al., 2023). This indicates that lactate, a differential metabolite mediated by EC dysfunction, may be a biomarker for the diagnosis and treatment of osteoporosis. Furthermore, BMSC from osteoporosis patients have reduced expression of histone H3K18la and collagen alpha-2(I) (COL1A2), cartilage oligomeric matrix protein (COMP), ectonucleotide pyrophosphatase/phosphodiesterase 1 (ENPP1) and transcription factor 7-like 2 (TCF7L2) of target gene, which inhibit osteoblast differentiation. Furthermore, lactate addition, and exercise restore the osteoporosis in mice. In summary, glycolysis within endothelial cells facilitates the differentiation of bone marrow-derived mesenchymal stem cells into osteoblasts *via* histone lactylation. Furthermore, physical exercise contributes to the partial mitigation of osteoporosis by elevating serum lactate concentrations (Wu et al., 2023). Overall, these results further elucidate that EC mediates osteoblast differentiation by promoting histone H3K18la. Lactate may be a biomarker for the diagnosis and treatment of osteoporosis.

### Role of Kla in age-related lung diseases

Idiopathic pulmonary fibrosis (IPF) is an age-related interstitial lung disease, mainly characterized by progressive scarring in lung tissues, reduced gas exchange, and impaired lung function (Chanda et al., 2019; Moss, Ryter & Rosas, 2022; Schafer et al., 2017; Wang et al., 2024). The pathological mechanism of IPF mainly involves fibroblast-to-myofibroblast transition (FMT) (Zhang et al., 2023a). Metabolic reprogramming plays an important regulatory role in FMT of IPF (Liu & Summer, 2019; Michaeloudes et al., 2020). Also, lactate level is significantly increased in lung extracts of mice and cellular IPF models. The glycolysis-induced increase in lactate levels promotes Kla of H3K18, which up-regulates pulmonary fibrosis-related genes, including ARG1, PDGFA, THBS1, and VEGFA, further exacerbating the occurrence of IPF (Cui et al., 2021). Additionally, studies have evaluated the specific mechanism of pulmonary fibrosis caused by Kla. For instance, Extracellular lactate from myofibroblasts increased global Kla and H3K18la through the lactate transporter MCT1. In alveolar epithelial cells (AECs), H3K18la enhanced Ythdf1 transcription. Elevated YTHDF1 and m6A-modified neuronal protein 3.1 (NREP) levels in AECs increased TGF-β1 secretion, promoting FMT. This study underscores the crosstalk between AECs and myofibroblasts *via* lactylation and m6A modification, highlighting H3K18la’s regulation of YTHDF1 in As-IPF progression, potentially aiding in therapeutic target discovery (Wang et al., 2024). Therefore, Kla upregulation in lung myofibroblasts through metabolic reprogramming is a potential pathogenesis of IPF, providing a potential target for IPF treatment.

### Role of Kla in age-related hepatic diseases

Aging is a key risk factor for chronic liver diseases, such as hepatitis, fibrosis, and cirrhosis (Stahl et al., 2018). Liver fibrosis (LF) involves massive deposition of intrahepatic ECM and occurs in most chronic liver diseases. The dysfunction of hepatic stellate cells (HSCs) and macrophages is closely related to the occurrence and development of LF with aging (Mahrouf-Yorgov et al., 2011). This can be because HSCs are quiescent under physiological conditions. HSCs are activated after stimulation by pathogenic factors to produce large amounts of ECM, leading to the occurrence of LF. Therefore, inhibiting HSC activation can improve LF (Iwaisako, Brenner & Kisseleva, 2012; Sherman, 2018; Zhang et al., 2024a). Aerobic glycolysis is a key feature of HSC activation. *In vitro* inhibition of glycolysis can significantly suppress HSC activation (Trivedi, Wang & Friedman, 2021). The enhancement of glycolysis increases lactate production during HSC activation, which enriches H3K18la at the α-SMA and COL1A1 promoters induced by HSC activation, thus controlling HSC activation and leading to LF. Additionally, lactate-modifying enzyme inhibitors reduce H3K18la modification levels, thus significantly inhibiting HSC activation and LF (Rho et al., 2023), suggesting that histone Kla plays a key role in HSC activation. However, the specific regulatory target genes are unknown. Additionally, HSPA12A, an atypical HSP70 family member, plays a protective role in liver injury by reducing glycolysis-driven HMGB1 lactylation and secretion in hepatocytes. Overexpression of HSPA12A in hepatocytes decreases HMGB1 levels, thereby inhibiting macrophage chemotaxis and inflammation. Conversely, knocking out HSPA12A enhances these harmful processes, but these effects are reversed by reducing HMGB1. Thus, HSPA12A acts as a key regulator in preventing liver injury by modulating HMGB1 lactylation. Targeting hepatocyte HSPA12A may have therapeutic potential in the management of LI/R injury in patients (Du et al., 2023). These findings indicate that Kla is closely related to the development of age-related hepatic diseases.

## Conclusions

Given the pivotal role of lactylation in disease pathology, modulating lactylation levels has emerged as a promising therapeutic strategy. However, the selection of therapeutic interventions must be tailored to the specific pathophysiological mechanisms of the disease. In the context of malignancies, neurodegenerative disorders (such as Alzheimer’s disease), and organ fibrosis (including pulmonary and hepatic fibrosis), lactylation typically exerts pathogenic effects. In these scenarios, the primary therapeutic objective is to reduce intracellular lactate levels and inhibit deleterious protein lactylation through the administration of glycolytic inhibitors (*e.g.*, 2-deoxy-D-glucose, 2-DG) or by targeting lactate-producing enzymes (*e.g.*, LDHA inhibitors).

Conversely, during the early reparative phases following myocardial infarction or in the management of osteoporosis, lactylation demonstrates protective or regenerative properties. For such conditions, therapeutic approaches aimed at elevating serum or local lactate concentrations—such as exogenous lactate supplementation, increased physical activity (exercise-induced hyperlactatemia), or the use of lactylation activators—can facilitate osteoblast differentiation or the expression of myocardial repair genes. This ‘disease-specific’ precision modulation strategy will be central to the future development of lactylation-targeted pharmacological agents.

## Supplemental Information

10.7717/peerj.20957/supp-1 Supplemental Information 1 PRISMA checklist

## Additional Information and Declarations

### Data Availability

The following information was supplied regarding data availability: This is a literature review.

## References

[ref-1] Andersen LL, Calatayud J, Nunez-Cortes R, Suso-Marti L, Polo-Lopez A, Lopez-Bueno R (2025). Association of distinct biomarker profiles with all-cause and cause-specific mortality in older adults: prospective cohort study across 12 countries. Nutrition, Metabolism and Cardiovascular Diseases.

[ref-2] Biegus J, Zymlinski R, Sokolski M, Gajewski P, Banasiak W, Ponikowski P (2019). Clinical, respiratory, haemodynamic, and metabolic determinants of lactate in heart failure. Kardiologia Polska.

[ref-3] Bonen A, Heynen M, Hatta H (2006). Distribution of monocarboxylate transporters MCT1-MCT8 in rat tissues and human skeletal muscle. Applied Physiology, Nutrition, and Metabolism.

[ref-4] Bosso G, Mercurio V, Diab N, Pagano A, Porta G, Allegorico E, Serra C, Guiotto G, Numis FG, Tocchetti CG, Schiraldi F (2021). Time-weighted lactate as a predictor of adverse outcome in acute heart failure. ESC Heart Failure.

[ref-5] Brown TP, Ganapathy V (2020). Lactate/GPR81 signaling and proton motive force in cancer: role in angiogenesis, immune escape, nutrition, and Warburg phenomenon. Pharmacology and Therapeutics.

[ref-6] Carrillo T, De Castro FR, Cuevas M, Caminero J, Cabrera P (1991). Allergy to limpet. Allergy.

[ref-7] Chai P, Zhao F, Jia R, Zhou X, Fan X (2025). Lactate/lactylation in ocular development and diseases. Trends in Molecular Medicine.

[ref-8] Chanda D, Otoupalova E, Smith SR, Volckaert T, De Langhe SP, Thannickal VJ (2019). Developmental pathways in the pathogenesis of lung fibrosis. Molecular Aspects of Medicine.

[ref-9] Chen AN, Luo Y, Yang YH, Fu JT, Geng XM, Shi JP, Yang J (2021). Lactylation, a novel metabolic reprogramming code: current status and prospects. Frontiers in Immunology.

[ref-10] Chen Y, Wu J, Zhai L, Zhang T, Yin H, Gao H, Zhao F, Wang Z, Yang X, Jin M, Huang B, Ding X, Li R, Yang J, He Y, Wang Q, Wang W, Kloeber JA, Li Y, Hao B, Zhang Y, Wang J, Tan M, Li K, Wang P, Lou Z, Yuan J (2024). Metabolic regulation of homologous recombination repair by MRE11 lactylation. Cell.

[ref-11] Cluntun AA, Badolia R, Lettlova S, Parnell KM, Shankar TS, Diakos NA, Olson KA, Taleb I, Tatum SM, Berg JA, Cunningham CN, Van Ry T, Bott AJ, Krokidi AT, Fogarty S, Skedros S, Swiatek WI, Yu X, Luo B, Merx S, Navankasattusas S, Cox JE, Ducker GS, Holland WL, McKellar SH, Rutter J, Drakos SG (2021). The pyruvate-lactate axis modulates cardiac hypertrophy and heart failure. Cell Metabolism.

[ref-12] Cui H, Xie N, Banerjee S, Ge J, Jiang D, Dey T, Matthews QL, Liu RM, Liu G (2021). Lung myofibroblasts promote macrophage profibrotic activity through lactate-induced histone lactylation. American Journal of Respiratory Cell and Molecular Biology.

[ref-13] Dai C, Li Q, May HI, Li C, Zhang G, Sharma G, Sherry AD, Malloy CR, Khemtong C, Zhang Y, Deng Y, Gillette TG, Xu J, Scadden DT, Wang ZV (2020). Lactate dehydrogenase a governs cardiac hypertrophic growth in response to hemodynamic stress. Cell Reports.

[ref-14] Dai W, Wu G, Liu K, Chen Q, Tao J, Liu H, Shen M (2023). Lactate promotes myogenesis *via* activating H3K9 lactylation-dependent up-regulation of Neu2 expression. Journal of Cachexia, Sarcopenia and Muscle.

[ref-15] De Leo A, Ugolini A, Veglia F (2024). Glucose-driven histone lactylation promotes the immunosuppressive activity of monocyte-derived macrophages in glioblastoma. The Journal of Immunology.

[ref-16] Deora AA, Philp N, Hu J, Bok D, Rodriguez-Boulan E (2005). Mechanisms regulating tissue-specific polarity of monocarboxylate transporters and their chaperone CD147 in kidney and retinal epithelia. Proceedings of the National Academy of Sciences of the United States of America.

[ref-17] Dong H, Zhang J, Zhang H, Han Y, Lu C, Chen C, Tan X, Wang S, Bai X, Zhai G, Tian S, Zhang T, Cheng Z, Li E, Xu L, Zhang K (2022). YiaC and CobB regulate lysine lactylation in *Escherichia coli*. Nature Communications.

[ref-18] Dong Q, Zhang Q, Yang X, Nai S, Du X, Chen L (2024). Glycolysis-stimulated esrrb lactylation promotes the self-renewal and extraembryonic endoderm stem cell differentiation of embryonic stem cells. International Journal of Molecular Sciences.

[ref-19] Du S, Zhang X, Jia Y, Peng P, Kong Q, Jiang S, Li Y, Li C, Ding Z, Liu L (2023). Hepatocyte HSPA12A inhibits macrophage chemotaxis and activation to attenuate liver ischemia/reperfusion injury *via* suppressing glycolysis-mediated HMGB1 lactylation and secretion of hepatocytes. Theranostics.

[ref-20] Eelen G, De Zeeuw P, Treps L, Harjes U, Wong BW, Carmeliet P (2018). Endothelial cell metabolism. Physiological Reviews.

[ref-21] Fagan AM, Henson RL, Li Y, Boerwinkle AH, Xiong C, Bateman RJ, Goate A, Ances BM, Doran E, Christian BT, Lai F, Rosas HD, Schupf N, Krinsky-McHale S, Silverman W, Lee JH, Klunk WE, Handen BL, Allegri RF, Chhatwal JP, Day GS, Graff-Radford NR, Jucker M, Levin J, Martins RN, Masters CL, Mori H, Mummery CJ, Niimi Y, Ringman JM, Salloway S, Schofield PR, Shoji M, Lott IT, Alzheimer’s Biomarker Consortium-Down S, and Dominantly Inherited Alzheimer N (2021). Comparison of CSF biomarkers in Down syndrome and autosomal dominant Alzheimer’s disease: a cross-sectional study. Lancet Neurology.

[ref-22] Fan H, Yang F, Xiao Z, Luo H, Chen H, Chen Z, Liu Q, Xiao Y (2023a). Lactylation: novel epigenetic regulatory and therapeutic opportunities. American Journal of Physiology, Endocrinology and Metabolism.

[ref-23] Fan M, Yang K, Wang X, Chen L, Gill PS, Ha T, Liu L, Lewis NH, Williams DL, Li C (2023b). Lactate promotes endothelial-to-mesenchymal transition *via* Snail1 lactylation after myocardial infarction. Science Advances.

[ref-24] Felmlee MA, Jones RS, Rodriguez-Cruz V, Follman KE, Morris ME (2020). Monocarboxylate transporters (SLC16): function, regulation, and role in health and disease. Pharmacological Reviews.

[ref-25] Fondy TP, Kaplan NO (1965). Structural and functional properties of the H and M subunits of lactic dehydrogenases. Annals of the New York Academy of Sciences.

[ref-26] Fu Y, Yu J, Li F, Ge S (2022). Oncometabolites drive tumorigenesis by enhancing protein acylation: from chromosomal remodelling to nonhistone modification. Journal of Experimental & Clinical Cancer Research.

[ref-27] Gao M, Zhang N, Liang W (2020). Systematic analysis of lysine lactylation in the plant fungal pathogen botrytis cinerea. Frontiers in Microbiology.

[ref-28] Global Nutrition Target Collaborators (2025). Global, regional, and national progress towards the 2030 global nutrition targets and forecasts to 2050: a systematic analysis for the Global Burden of Disease Study 2021. Lancet.

[ref-29] Gong F, Miller KM (2019). Histone methylation and the DNA damage response. Mutation Research/Reviews in Mutation Research.

[ref-30] Goumans MJ, Ten Dijke P (2018). TGF-*β* signaling in control of cardiovascular function. Cold Spring Harbor Perspectives in Biology.

[ref-31] Grunstein M (1997). Histone acetylation in chromatin structure and transcription. Nature.

[ref-32] Gu J, Zhou J, Chen Q, Xu X, Gao J, Li X, Shao Q, Zhou B, Zhou H, Wei S, Wang Q, Liang Y, Lu L (2022). Tumor metabolite lactate promotes tumorigenesis by modulating MOESIN lactylation and enhancing TGF-beta signaling in regulatory T cells. Cell Reports.

[ref-33] Haege ER, Huang HC, Huang CC (2021). Identification of lactate as a cardiac protectant by inhibiting inflammation and cardiac hypertrophy using a zebrafish acute heart failure model. Pharmaceuticals.

[ref-34] Hagihara H, Shoji H, Otabi H, Toyoda A, Katoh K, Namihira M, Miyakawa T (2021). Protein lactylation induced by neural excitation. Cell Reports.

[ref-35] Halestrap AP (2013). The SLC16 gene family - structure, role and regulation in health and disease. Molecular Aspects of Medicine.

[ref-36] Hilgendorf I, Gerhardt LM, Tan TC, Winter C, Holderried TA, Chousterman BG, Iwamoto Y, Liao R, Zirlik A, Scherer-Crosbie M, Hedrick CC, Libby P, Nahrendorf M, Weissleder R, Swirski FK (2014). Ly-6Chigh monocytes depend on Nr4a1 to balance both inflammatory and reparative phases in the infarcted myocardium. Circulation Research.

[ref-37] Hu X, Huang X, Yang Y, Sun Y, Zhao Y, Zhang Z, Qiu D, Wu Y, Wu G, Lei L (2024). Dux activates metabolism-lactylation-MET network during early iPSC reprogramming with Brg1 as the histone lactylation reader. Nucleic Acids Research.

[ref-38] Hu Y, Mai W, Chen L, Cao K, Zhang B, Zhang Z, Liu Y, Lou H, Duan S, Gao Z (2020). mTOR-mediated metabolic reprogramming shapes distinct microglia functions in response to lipopolysaccharide and ATP. Glia.

[ref-39] Huang ZW, Zhang XN, Zhang L, Liu LL, Zhang JW, Sun YX, Xu JQ, Liu Q, Long ZJ (2023). STAT5 promotes PD-L1 expression by facilitating histone lactylation to drive immunosuppression in acute myeloid leukemia. Signal Transduction and Targeted Therapy.

[ref-40] Hunter T (2007). The age of crosstalk: phosphorylation, ubiquitination, and beyond. Molecular Cell.

[ref-41] Iizuka K, Machida T, Hirafuji M (2014). Extracellular MCT4 is a possible indicator for skeletal muscle MHC fiber type change. Annals of Clinical and Laboratory Science.

[ref-42] Iwaisako K, Brenner DA, Kisseleva T (2012). What’s new in liver fibrosis? The origin of myofibroblasts in liver fibrosis. Journal of Gastroenterology and Hepatology.

[ref-43] Jeurissen D, Shushruth S, El-Shamayleh Y, Horwitz GD, Shadlen MN (2022). Deficits in decision-making induced by parietal cortex inactivation are compensated at two timescales. Neuron.

[ref-44] Ju J, Zhang H, Lin M, Yan Z, An L, Cao Z, Geng D, Yue J, Tang Y, Tian L, Chen F, Han Y, Wang W, Zhao S, Shi J, Zhou Z (2024). The alanyl-tRNA synthetase AARS1 moonlights as a lactyl-transferase to promote YAP signaling in gastric cancer. The Journal of Clinical Investigation.

[ref-45] Karwi QG, Zhang L, Altamimi TR, Wagg CS, Patel V, Uddin GM, Joerg AR, Padwal RS, Johnstone DE, Sharma A, Oudit GY, Lopaschuk GD (2019). Weight loss enhances cardiac energy metabolism and function in heart failure associated with obesity. Diabetes, Obesity and Metabolism.

[ref-46] Kawakami S, Johmura Y, Nakanishi M (2024). Intracellular acidification and glycolysis modulate inflammatory pathway in senescent cells. Journal of Biochemistry.

[ref-47] Kirat D, Kato S (2015). The monocarboxylate transporters exist in the cattle endocrine pancreas. Histochemistry and Cell Biology.

[ref-48] Kirat D, Sallam K, Hayashi H, Miyasho T, Kato S (2009). Presence of ten isoforms of monocarboxylate transporter (MCT) family in the bovine adrenal gland. Molecular and Cellular Endocrinology.

[ref-49] Kokudo T, Suzuki Y, Yoshimatsu Y, Yamazaki T, Watabe T, Miyazono K (2008). Snail is required for TGF*β*-induced endothelial-mesenchymal transition of embryonic stem cell-derived endothelial cells. Journal of Cell Science.

[ref-50] Koprinarova M, Schnekenburger M, Diederich M (2016). Role of histone acetylation in cell cycle regulation. Current Topics in Medicinal Chemistry.

[ref-51] Kotha S, Sahu R, Yadav AC, Sharma P, Kumar B, Reddy SK, Rao KV (2024). Noncovalent synthesis of homo and hetero-architectures of supramolecular polymers *via* secondary nucleation. Nature Communications.

[ref-52] Kovacic JC, Dimmeler S, Harvey RP, Finkel T, Aikawa E, Krenning G, Baker AH (2019). Endothelial to mesenchymal transition in cardiovascular disease: JACC state-of-the-art review. Journal of the American College of Cardiology.

[ref-53] Li S, Nguyen TT, Bonanno JA (2014). CD147 required for corneal endothelial lactate transport. Investigative Ophthalmology and Visual Science.

[ref-54] Li P, Wu M, Wang R, Zhang G, Kang L, Guan H, Ji M (2025). Spatial alteration of metabolites in diabetic cortical cataracts: new insight into lactate. Experimental Eye Research.

[ref-55] Li X, Yang N, Wu Y, Wang X, Sun J, Liu L, Zhang F, Gong Y, Zhang Y, Li X, Du D, Ding B (2022a). Hypoxia regulates fibrosis-related genes *via* histone lactylation in the placentas of patients with preeclampsia. Journal of Hypertension.

[ref-56] Li X, Yang Y, Zhang B, Lin X, Fu X, An Y, Zou Y, Wang JX, Wang Z, Yu T (2022b). Lactate metabolism in human health and disease. Signal Transduction and Targeted Therapy.

[ref-57] Li J, Zeng G, Zhang Z, Wang Y, Shao M, Li C, Lu Z, Zhao Y, Zhang F, Ding W (2024a). Urban airborne PM_(2.5)_ induces pulmonary fibrosis through triggering glycolysis and subsequent modification of histone lactylation in macrophages. Ecotoxicology and Environmental Safety.

[ref-58] Li Q, Zhang F, Wang H, Tong Y, Fu Y, Wu K, Li J, Wang C, Wang Z, Jia Y, Chen R, Wu Y, Cui R, Wu Y, Qi Y, Qu K, Liu C, Zhang J (2024b). NEDD4 lactylation promotes APAP induced liver injury through Caspase11 dependent non-canonical pyroptosis. International Journal of Biological Sciences.

[ref-59] Liguori C, Stefani A, Sancesario G, Sancesario GM, Marciani MG, Pierantozzi M (2015). CSF lactate levels, tau proteins, cognitive decline: a dynamic relationship in Alzheimer’s disease. Journal of Neurology, Neurosurgery and Psychiatry.

[ref-60] Lin X, Lei Y, Pan M, Hu C, Xie B, Wu W, Su J, Li Y, Tan Y, Wei X, Xue Z, Xu R, Deng HDi M, Liu S, Yang X, Qu J, Chen W, Zhou X, Zhao F (2024). Augmentation of scleral glycolysis promotes myopia through histone lactylation. Cell Metabolism.

[ref-61] Liu G, Summer R (2019). Cellular metabolism in lung health and disease. Annual Review of Physiology.

[ref-62] Lu C, Coradin M, Porter EG, Garcia BA (2021). Accelerating the field of epigenetic histone modification through mass spectrometry-based approaches. Molecular & Cellular Proteomics.

[ref-63] Luz MC, Perez MM, Azzalis LA, Sousa LV, Adami F, Fonseca FL, Alves BD (2017). Evaluation of MCT1, MCT4 and CD147 genes in peripheral blood cells of breast cancer patients and their potential use as diagnostic and prognostic markers. International Journal of Molecular Sciences.

[ref-64] Ma W, Jia K, Cheng H, Xu H, Li Z, Zhang H, Xie H, Zhuang L, Wang Z, Cui Y, Sun H, Yi L, Chen Z, Duan S, Sano M, Fukuda K, Lu L, Gao F, Zhang R, Yan X (2024). Orphan nuclear receptor NR4A3 promotes vascular calcification *via* histone lactylation. Circulation Research.

[ref-65] Mahrouf-Yorgov M, Collin de l’Hortet A, Cosson C, Slama A, Abdoun E, Guidotti JE, Fromenty B, Mitchell C, Gilgenkrantz H (2011). Increased susceptibility to liver fibrosis with age is correlated with an altered inflammatory response. Rejuvenation Research.

[ref-66] Mao Y, Zhang J, Zhou Q, He X, Zheng Z, Wei Y, Zhou K, Lin Y, Yu H, Zhang H, Zhou Y, Lin P, Wu B, Yuan Y, Zhao J, Xu W, Zhao S (2024). Hypoxia induces mitochondrial protein lactylation to limit oxidative phosphorylation. Cell Research.

[ref-67] Markert CL, Shaklee JB, Whitt GS (1975). Evolution of a gene. Multiple genes for LDH isozymes provide a model of the evolution of gene structure, function and regulation. Science.

[ref-68] Martin SS, Aday AW, Almarzooq ZI, Anderson CAM, Arora P, Avery CL, Baker-Smith CM, Gibbs BBarone, Beaton AZ, Boehme AK, Commodore-Mensah Y, Currie ME, Elkind MSV, Evenson KR, Generoso G, Heard DG, Hiremath S, Johansen MC, Kalani R, Kazi DS, Ko D, Liu J, Magnani JW, Michos ED, Mussolino ME, Navaneethan SD, Parikh NI, Perman SM, Poudel R, Rezk-Hanna M, Roth GA, Shah NS, St-Onge MP, Thacker EL, Tsao CW, Urbut SM, Van Spall HGC, Voeks JH, Wang NY, Wong ND, Wong SS, Yaffe K, Palaniappan LP, American Heart Association Council on Epidemiology and Prevention Statistics Committee and Stroke Statistics Subcommittee (2024). 2024 Heart disease and stroke statistics: a report of US and global data from the american heart association. Circulation.

[ref-69] Mathew M, Nguyen NT, Bhutia YD, Sivaprakasam S, Ganapathy V (2024). Correction: Mathew et al. Metabolic signature of warburg effect in cancer: an effective and obligatory interplay between nutrient transporters and catabolic/anabolic pathways to promote tumor growth. Cancers.

[ref-70] Mawuenyega KG, Sigurdson W, Ovod V, Munsell L, Kasten T, Morris JC, Yarasheski KE, Bateman RJ (2010). Decreased clearance of CNS beta-amyloid in Alzheimer’s disease. Science.

[ref-71] McIntosh A, Mela V, Harty C, Minogue AM, Costello DA, Kerskens C, Lynch MA (2019). Iron accumulation in microglia triggers a cascade of events that leads to altered metabolism and compromised function in APP/PS1 mice. Brain Pathology.

[ref-72] Michaeloudes C, Bhavsar PK, Mumby S, Xu B, Hui CKM, Chung KF, Adcock IM (2020). Role of metabolic reprogramming in pulmonary innate immunity and its impact on lung diseases. Journal of Innate Immunity.

[ref-73] Miller MB, Huang AY, Kim J, Zhou Z, Kirkham SL, Maury EA, Ziegenfuss JS, Reed HC, Neil JE, Rento L, Ryu SC, Ma CC, Luquette LJ, Ames HM, Oakley DH, Frosch MP, Hyman BT, Lodato MA, Lee EA, Walsh CA (2022). Somatic genomic changes in single Alzheimer’s disease neurons. Nature.

[ref-74] Moreno-Yruela C, Zhang D, Wei W, Baek M, Liu W, Gao J, Dankova D, Nielsen AL, Bolding JE, Yang L, Jameson ST, Wong J, Olsen CA, Zhao Y (2022). Class I histone deacetylases (HDAC1-3) are histone lysine delactylases. Science Advances.

[ref-75] Morrison SJ, Scadden DT (2014). The bone marrow niche for haematopoietic stem cells. Nature.

[ref-76] Moss BJ, Ryter SW, Rosas IO (2022). Pathogenic mechanisms underlying idiopathic pulmonary fibrosis. Annual Review of Pathology: Mechanisms of Disease.

[ref-77] Mrozikiewicz AE, Kurzawinska G, Walczak M, Skrzypczak-Zielinska M, Ozarowski M, Jedrzejczak P (2024). Up-regulated mRNA expression of VEGFA receptors (*FLT1* and *KDR*) in placentas after assisted reproductive technology fertilization. Journal of Applied Genetics.

[ref-78] Murashige D, Jang C, Neinast M, Edwards JJ, Cowan A, Hyman MC, Rabinowitz JD, Frankel DS, Arany Z (2020). Comprehensive quantification of fuel use by the failing and nonfailing human heart. Science.

[ref-79] Nahrendorf M, Swirski FK (2016). Innate immune cells in ischaemic heart disease: does myocardial infarction beget myocardial infarction?. European Heart Journal.

[ref-80] Nalos M, Leverve X, Huang S, Weisbrodt L, Parkin R, Seppelt I, Ting I, McLean A (2014). Half-molar sodium lactate infusion improves cardiac performance in acute heart failure: a pilot randomised controlled clinical trial. Critical Care.

[ref-81] Niu Z, Chen C, Wang S, Lu C, Wu Z, Wang A, Mo J, Zhang J, Han Y, Yuan Y, Zhang Y, Zang Y, He C, Bai X, Tian S, Zhai G, Wu X, Zhang K (2024). HBO1 catalyzes lysine lactylation and mediates histone H3K9la to regulate gene transcription. Nature Communications.

[ref-82] Pan L, Feng F, Wu J, Fan S, Han J, Wang S, Yang L, Liu W, Wang C, Xu K (2022a). Demethylzeylasteral targets lactate by inhibiting histone lactylation to suppress the tumorigenicity of liver cancer stem cells. Pharmacological Research.

[ref-83] Pan RY, He L, Zhang J, Liu X, Liao Y, Gao J, Liao Y, Yan Y, Li Q, Zhou X, Cheng J, Xing Q, Guan F, Zhang J, Sun L, Yuan Z (2022b). Positive feedback regulation of microglial glucose metabolism by histone H4 lysine 12 lactylation in Alzheimer’s disease. Cell Metabolism.

[ref-84] Pandkar MR, Sinha S, Samaiya A, Shukla S (2023). Oncometabolite lactate enhances breast cancer progression by orchestrating histone lactylation-dependent c-Myc expression. Translational Oncology.

[ref-85] Potente M, Carmeliet P (2017). The link between angiogenesis and endothelial metabolism. Annual Review of Physiology.

[ref-86] Rho H, Terry AR, Chronis C, Hay N (2023). Hexokinase 2-mediated gene expression *via* histone lactylation is required for hepatic stellate cell activation and liver fibrosis. Cell Metabolism.

[ref-87] Sacco RL, Kargman DE, Gu Q, Zamanillo MC (1995). Race-ethnicity and determinants of intracranial atherosclerotic cerebral infarction. Stroke.

[ref-88] Sadakierska-Chudy A, Filip M (2015). A comprehensive view of the epigenetic landscape. Part II: histone post-translational modification, nucleosome level, and chromatin regulation by ncRNAs. Neurotoxicity Research.

[ref-89] Sanghani-Kerai A, Osagie-Clouard L, Blunn G, Coathup M (2018). The influence of age and osteoporosis on bone marrow stem cells from rats. Bone & Joint Research.

[ref-90] Schafer MJ, White TA, Iijima K, Haak AJ, Ligresti G, Atkinson EJ, Oberg AL, Birch J, Salmonowicz H, Zhu Y, Mazula DL, Brooks RW, Fuhrmann-Stroissnigg H, Pirtskhalava T, Prakash YS, Tchkonia T, Robbins PD, Aubry MC, Passos JF, Kirkland JL, Tschumperlin DJ, Kita H, LeBrasseur NK (2017). Cellular senescence mediates fibrotic pulmonary disease. Nature Communications.

[ref-91] Schaft D, Roguev A, Kotovic KM, Shevchenko A, Sarov M, Shevchenko A, Neugebauer KM, Stewart AF (2003). The histone 3 lysine 36 methyltransferase, SET2, is involved in transcriptional elongation. Nucleic Acids Research.

[ref-92] Sherman MH (2018). Stellate cells in tissue repair, inflammation, and cancer. Annual Review of Cell and Developmental Biology.

[ref-93] Sivaprakasam S, Bhutia YD, Yang S, Ganapathy V (2017). Short-chain fatty acid transporters: role in colonic homeostasis. Comprehensive Physiology.

[ref-94] Stahl EC, Haschak MJ, Popovic B, Brown BN (2018). Macrophages in the aging liver and age-related liver disease. Frontiers in Immunology.

[ref-95] Sun Y, Chen Y, Peng T (2022). A bioorthogonal chemical reporter for the detection and identification of protein lactylation. Chemical Science.

[ref-96] Sun R, Peng M, Xu P, Huang F, Xie Y, Li J, Hong Y, Guo H, Liu Q, Zhu W (2020). Low-density lipoprotein receptor (LDLR) regulates NLRP3-mediated neuronal pyroptosis following cerebral ischemia/reperfusion injury. Journal of Neuroinflammation.

[ref-97] Tanaka H, Sueyoshi K, Nishino M, Ishida M, Fukunaga R, Abe H (1993). Silent brain infarction and coronary artery disease in Japanese patients. Archives of Neurology.

[ref-98] Tejera D, Mercan D, Sanchez-Caro JM, Hanan M, Greenberg D, Soreq H, Latz E, Golenbock D, Heneka MT (2019). Systemic inflammation impairs microglial Abeta clearance through NLRP3 inflammasome. EMBO Journal.

[ref-99] Tian Q, Zhou LQ (2022). Lactate activates germline and cleavage embryo genes in mouse embryonic stem cells. Cells.

[ref-100] Tombor LS, John D, Glaser SF, Luxan G, Forte E, Furtado M, Rosenthal N, Baumgarten N, Schulz MH, Wittig J, Rogg EM, Manavski Y, Fischer A, Muhly-Reinholz M, Klee K, Looso M, Selignow C, Acker T, Bibli SI, Fleming I, Patrick R, Harvey RP, Abplanalp WT, Dimmeler S (2021). Single cell sequencing reveals endothelial plasticity with transient mesenchymal activation after myocardial infarction. Nature Communications.

[ref-101] Trivedi P, Wang S, Friedman SL (2021). The power of plasticity-metabolic regulation of hepatic stellate cells. Cell Metabolism.

[ref-102] Wang N, Wang W, Wang X, Mang G, Chen J, Yan X, Tong Z, Yang Q, Wang M, Chen L, Sun P, Yang Y, Cui J, Yang M, Zhang Y, Wang D, Wu J, Zhang M, Yu B (2022). Histone lactylation boosts reparative gene activation post-myocardial infarction. Circulation Research.

[ref-103] Wang P, Xie D, Xiao T, Cheng C, Wang D, Sun J, Wu M, Yang Y, Zhang A, Liu Q (2024). H3K18 lactylation promotes the progression of arsenite-related idiopathic pulmonary fibrosis *via* YTHDF1/m6A/NREP. Journal of Hazardous Materials.

[ref-104] Wei L, Yang X, Wang J, Wang Z, Wang Q, Ding Y, Yu A (2023). H3K18 lactylation of senescent microglia potentiates brain aging and Alzheimer’s disease through the NFκB signaling pathway. Journal of Neuroinflammation.

[ref-105] Wen M, Jin Y, Zhang H, Sun X, Kuai Y, Tan W (2019). Proteomic analysis of rat cerebral cortex in the subacute to long-term phases of focal cerebral ischemia-reperfusion injury. Journal of Proteome Research.

[ref-106] Wu S, Chen J (2026). Is age-related myelinodegenerative change an initial risk factor of neurodegenerative diseases?. Neural Regeneration Research.

[ref-107] Wu J, Hu M, Jiang H, Ma J, Xie C, Zhang Z, Zhou X, Zhao J, Tao Z, Meng Y, Cai Z, Song T, Zhang C, Gao R, Cai C, Song H, Gao Y, Lin T, Wang C, Zhou X (2023). Endothelial cell-derived lactate triggers bone mesenchymal stem cell histone lactylation to attenuate osteoporosis. Advanced Science.

[ref-108] Wu Y, Ke C, Song Z, Zhu H, Guo H, Sun H, Liu M (2024). Fluorescence and colorimetric dual-mode multienzyme cascade nanoplatform based on CuNCs/FeMn-ZIF-8/PCN for detection of sarcosine. Analyst.

[ref-109] Xie B, Zhang M, Li J, Cui J, Zhang P, Liu F, Wu Y, Deng W, Ma J, Li X, Pan B, Zhang B, Zhang H, Luo A, Xu Y, Li M, Pu Y (2024). KAT8-catalyzed lactylation promotes eEF1A2-mediated protein synthesis and colorectal carcinogenesis. Proceedings of the National Academy of Sciences of the United States of America.

[ref-110] Xiong J, He J, Zhu J, Pan J, Liao W, Ye H, Wang H, Song Y, Du Y, Cui B, Xue M, Zheng W, Kong X, Jiang K, Ding K, Lai L, Wang Q (2022). Lactylation-driven METTL3-mediated RNA m6A modification promotes immunosuppression of tumor-infiltrating myeloid cells. Molecular Cell.

[ref-111] Xu H, Li L, Wang S, Wang Z, Qu L, Wang C, Xu K (2023). Royal jelly acid suppresses hepatocellular carcinoma tumorigenicity by inhibiting H3 histone lactylation at H3K9la and H3K14la sites. Phytomedicine.

[ref-112] Xu H, Wu M, Ma X, Huang W, Xu Y (2021). Function and mechanism of novel histone posttranslational modifications in health and disease. BioMed Research International.

[ref-113] Yang K, Fan M, Wang X, Xu J, Wang Y, Tu F, Gill PS, Ha T, Liu L, Williams DL, Li C (2022b). Lactate promotes macrophage HMGB1 lactylation, acetylation, and exosomal release in polymicrobial sepsis. Cell Death and Differentiation.

[ref-114] Yang J, Luo L, Zhao C, Li X, Wang Z, Zeng Z, Yang X, Zheng X, Jie H, Kang L, Li S, Liu S, Zhou C, Liu H (2022a). A positive feedback loop between inactive VHL-triggered histone lactylation and PDGFRbeta signaling drives clear cell renal cell carcinoma progression. International Journal of Biological Sciences.

[ref-115] Yang C, Pan RY, Guan F, Yuan Z (2024). Lactate metabolism in neurodegenerative diseases. Neural Regeneration Research.

[ref-116] Yang H, Yang S, He J, Li W, Zhang A, Li N, Zhou G, Sun B (2023). Glucose transporter 3 (GLUT3) promotes lactylation modifications by regulating lactate dehydrogenase A (LDHA) in gastric cancer. Cancer Cell International.

[ref-117] Yao X, Li C (2023). Lactate dehydrogenase A mediated histone lactylation induced the pyroptosis through targeting HMGB1. Metabolic Brain Disease.

[ref-118] Yin X, Li M, Wang Y, Zhao G, Yang T, Zhang Y, Guo J, Meng T, Du R, Li H, Wang Z, Zhang J, He Q (2023). Herbal medicine formula Huazhuo Tiaozhi granule ameliorates dyslipidaemia *via* regulating histone lactylation and miR-155-5p biogenesis. Clinical Epigenetics.

[ref-119] Yu J, Chai P, Xie M, Ge S, Ruan J, Fan X, Jia R (2021). Histone lactylation drives oncogenesis by facilitating m(6)A reader protein YTHDF2 expression in ocular melanoma. Genome Biology.

[ref-120] Yu W, Kong Q, Jiang S, Li Y, Wang Z, Mao Q, Zhang X, Liu Q, Zhang P, Li Y, Li C, Ding Z, Liu L (2024). HSPA12A maintains aerobic glycolytic homeostasis and Histone3 lactylation in cardiomyocytes to attenuate myocardial ischemia/reperfusion injury. JCI Insight.

[ref-121] Yue Y, Dong S, Wu Z, Dong Y, Chen Q, Wang H, Liu C, Yang D (2025). Identify of blood glucose metabolism regulation pathways and related proteins in the db/db mouse model through iTRAQ quantitative mass spectrometry. Acta Diabetologica.

[ref-122] Zhang Y, Huang Z, Han W, Wu J, Li S, Qin T, Zhang C, Shi M, Han S, Gao B, Jin S, Xiao Y, Xu K, Ye W (2024b). Glutamine suppresses senescence and promotes autophagy through glycolysis inhibition-mediated AMPK*α* lactylation in intervertebral disc degeneration. Communications Biology.

[ref-123] Zhang N, Jiang N, Yu L, Guan T, Sang X, Feng Y, Chen R, Chen Q (2021a). Protein lactylation critically regulates energy metabolism in the protozoan parasite Trypanosoma brucei. Frontiers in Cell and Developmental Biology.

[ref-124] Zhang R, Liu Y, Zhang C, Ma M, Li S, Hong Y (2021b). Salt-inducible kinase 2 regulates energy metabolism in rats with cerebral ischemia-reperfusion. Zhejiang Da Xue Xue Bao Yi Xue Ban.

[ref-125] Zhang Y, Peng Q, Zheng J, Yang Y, Zhang X, Ma A, Qin Y, Qin Z, Zheng X (2023d). The function and mechanism of lactate and lactylation in tumor metabolism and microenvironment. Genes & Diseases.

[ref-126] Zhang D, Tang Z, Huang H, Zhou G, Cui C, Weng Y, Liu W, Kim S, Lee S, Perez-Neut M, Ding J, Czyz D, Hu R, Ye Z, He M, Zheng YG, Shuman HA, Dai L, Ren B, Roeder RG, Becker L, Zhao Y (2019). Metabolic regulation of gene expression by histone lactylation. Nature.

[ref-127] Zhang T, Wang C, Song A, Lei X, Li G, Sun H, Wang X, Geng Z, Shu G, Deng X (2024a). Water extract of earthworms mitigates mouse liver fibrosis by potentiating hepatic LKB1/Nrf2 axis to inhibit HSC activation and hepatocyte death. Journal of Ethnopharmacology.

[ref-128] Zhang W, Xu L, Yu Z, Zhang M, Liu J, Zhou J (2023c). Inhibition of the glycolysis prevents the cerebral infarction progression through decreasing the lactylation levels of LCP1. Molecular Biotechnology.

[ref-129] Zhang N, Zhang Y, Xu J, Wang P, Wu B, Lu S, Lu X, You S, Huang X, Li M, Zou Y, Liu M, Zhao Y, Sun G, Wang W, Geng D, Liu J, Cao L, Sun Y (2023b). *α*-myosin heavy chain lactylation maintains sarcomeric structure and function and alleviates the development of heart failure. Cell Research.

[ref-130] Zhang H, Zhou Y, Wen D, Wang J (2023a). Noncoding RNAs: master regulator of fibroblast to myofibroblast transition in fibrosis. International Journal of Molecular Sciences.

[ref-131] Zhao X, Li S, Mo Y, Li R, Huang S, Zhang A, Ni X, Dai Q, Wang J (2021). DCA protects against oxidation injury attributed to cerebral ischemia-reperfusion by regulating glycolysis through PDK2-PDH-Nrf2 axis. Oxidative Medicine and Cellular Longevity.

[ref-132] Zhao W, Yu H, Liu X, Wang T, Yao Y, Zhou Q, Zheng X, Tan F (2022). Systematic identification of the lysine lactylation in the protozoan parasite *Toxoplasma gondii*. Parasit Vectors.

[ref-133] Zhou Y, Yan J, Huang H, Liu L, Ren L, Hu J, Jiang X, Zheng Y, Xu L, Zhong F, Li X (2024). The m^(6)^A reader IGF2BP2 regulates glycolytic metabolism and mediates histone lactylation to enhance hepatic stellate cell activation and liver fibrosis. Cell Death & Disease.

[ref-134] Zhou Y, Zhu W, Yin X, Zeng M, Wang J (2025). Lactate metabolism and lactylation in ocular diseases. Experimental Eye Research.

[ref-135] Zong Z, Ren J, Yang B, Zhang L, Zhou F (2025). Emerging roles of lysine lactyltransferases and lactylation. Nature Cell Biology.

[ref-136] Zong Z, Xie F, Wang S, Wu X, Zhang Z, Yang B, Zhou F (2024). Alanyl-tRNA synthetase, AARS1, is a lactate sensor and lactyltransferase that lactylates p53 and contributes to tumorigenesis. Cell.

[ref-137] Zymlinski R, Biegus J, Sokolski M, Siwolowski P, Nawrocka-Millward S, Todd J, Jankowska EA, Banasiak W, Cotter G, Cleland JG, Ponikowski P (2018). Increased blood lactate is prevalent and identifies poor prognosis in patients with acute heart failure without overt peripheral hypoperfusion. European Journal of Heart Failure.

